# Reducing mitochondrial ribosomal gene expression does not alter metabolic health or lifespan in mice

**DOI:** 10.1038/s41598-023-35196-3

**Published:** 2023-05-24

**Authors:** Kim Reid, Eileen G. Daniels, Goutham Vasam, Rashmi Kamble, Georges E. Janssens, Iman M. Hu, Alexander E. Green, Riekelt H. Houtkooper, Keir J. Menzies

**Affiliations:** 1 Department of Biology and Ottawa Institute of Systems Biology, University of Ottawa, 30 Marie Curie Private, Ottawa, ON Canada; 2 Laboratory Genetic Metabolic Diseases, Amsterdam UMC Location University of Amsterdam, Meibergdreef 9, 1105 AZ Amsterdam, The Netherlands; 3 Amsterdam Gastroenterology Endocrinology and Metabolism Institute, Amsterdam, The Netherlands; 4 Interdisciplinary School of Health Sciences, University of Ottawa, 451 Smyth Road, Ottawa, ON K1H 8M5 Canada; 5 Present Address: Department of Biochemistry, Microbiology and Immunology, Faculty of Medicine, Ottawa Institute of Systems Biology and the Éric Poulin Centre for Neuromuscular Disease, University of Ottawa, Ottawa, ON Canada; 6 Amsterdam Cardiovascular Sciences Institute, Amsterdam, The Netherlands

**Keywords:** Cell biology, Energy metabolism

## Abstract

Maintaining mitochondrial function is critical to an improved healthspan and lifespan. Introducing mild stress by inhibiting mitochondrial translation invokes the mitochondrial unfolded protein response (UPR^mt^) and increases lifespan in several animal models. Notably, lower mitochondrial ribosomal protein (MRP) expression also correlates with increased lifespan in a reference population of mice. In this study, we tested whether partially reducing the gene expression of a critical MRP, *Mrpl54*, reduced mitochondrial DNA-encoded protein content, induced the UPR^mt^, and affected lifespan or metabolic health using germline heterozygous *Mrpl54* mice. Despite reduced *Mrpl54* expression in multiple organs and a reduction in mitochondrial-encoded protein expression in myoblasts, we identified few significant differences between male or female *Mrpl54*^+/−^ and wild type mice in initial body composition, respiratory parameters, energy intake and expenditure, or ambulatory motion. We also observed no differences in glucose or insulin tolerance, treadmill endurance, cold tolerance, heart rate, or blood pressure. There were no differences in median life expectancy or maximum lifespan. Overall, we demonstrate that genetic manipulation of *Mrpl54* expression reduces mitochondrial-encoded protein content but is not sufficient to improve healthspan in otherwise healthy and unstressed mice.

## Introduction

Since 1972, mitochondria have been implicated in the aging process^1–4^, initially as a source of free radicals in the mitochondrial theory of aging, and more recently as an indicator where reduced mitochondrial respiratory chain efficiency is considered a cellular hallmark of aging^5,6^. This is because mitochondrial dysfunction: (1) occurs during normal aging^7–9^; (2) is implicated in age-related pathologies such as type 2 diabetes, cardiovascular disease, and cancer^10–12^; and (3) aggravation of mitochondrial dysfunction accelerates aging^13^. If mitochondrial dysfunction contributes to aging, then improving mitochondrial function should slow normal aging^14^. Although seemingly contradictory, mitochondrial stress early in development maintains mitochondrial function with age in several animal models^15–19^. This stress triggers a compensatory beneficial response—i.e., a mitochondrial hormetic (mitohormetic) response, which is suggested to improve healthspan and/or lifespan^6,20^. If mild mitochondrial stress improves healthspan or extends lifespan in mammals, then this opens new possibilities in human healthspan and longevity research.

Specifically, mitochondrial translational stress is emerging as a promising mitohormetic strategy^15,21,22^. The mitochondrial ribosome (mitoribosome), which consists of ~ 80 mitoribosomal proteins (MRPs)^23^, conducts mitochondrial translation to produce essential electron transport chain (ETC) complex protein subunits encoded by mitochondrial DNA. Previously, we showed that partial knockdown of any individual MRP, and the subsequent disruption of mitochondrial translation, improved metabolic health and extended the lifespan of *Caenorhabditis elegans (C. elegans)*^15^. Reducing the expression of a single MRP induced a mitonuclear protein imbalance, a stoichiometric imbalance between nuclear-(nDNA) and mitochondrial-DNA (mtDNA) encoded ETC subunits. This imbalance subsequently activated the mitochondrial unfolded protein response (UPR^mt^)—a nuclear transcriptional response that maintains or restores mitochondrial proteostasis^24–26^. Strikingly, the variation in expression of MRP genes amongst recombinant-inbred BXD mouse strains^27,28^, e.g., *Mrps5* expression, inversely correlated with a ~ 250-day increase in lifespan^15^. Further, fibroblast cell cultures from long-lived Snell dwarf mice had greater expression of UPR^mt^ markers, including elevated 60 kDa mitochondrial heat shock protein (HSP60 or HSPD1)^29^. Therefore, chronic disruption of mitochondrial translation and induction of the UPR^mt^ may promote both longevity and an improved healthspan via mitohormesis^15,30–35^.

The observation that the reduction of MRP expression and consequent induction of mitonuclear imbalance induced the UPR^mt^ and promoted longevity across species led us to a general hypothesis: partial disruption of a critical *Mrp* gene in a mouse model would result in improved metabolic health with age and/or an increased lifespan. To test this hypothesis, we engineered a whole-body mouse model heterozygous for the *Mrpl54* gene (*Mrpl54*^+*/−*^). The MRPL54 protein was selected because: (1) reduced expression of *Mrpl54* invokes the UPR^mt^ and extends *C. elegans* lifespan^15^; (2) MRPL54 is evolutionarily conserved between species^36^; and (3) MRPL54 is essential to both recruitment of essential factors for mitochondrial translation^37,38^ and ETC function^39^. To assess metabolic health with age, we subjected both male and female *Mrpl54*^+/−^ and *Mrpl54*^+*/*+^ (wildtype, WT) littermate mice to in vivo metabolic phenotyping at 6-, 18-, and 24-months of age. In parallel, we conducted a longevity study to assess the effect of mitochondrial stress on life expectancy and lifespan.

## Results

### Generation of Mrpl54^+/−^ mice

**Figure 1:**
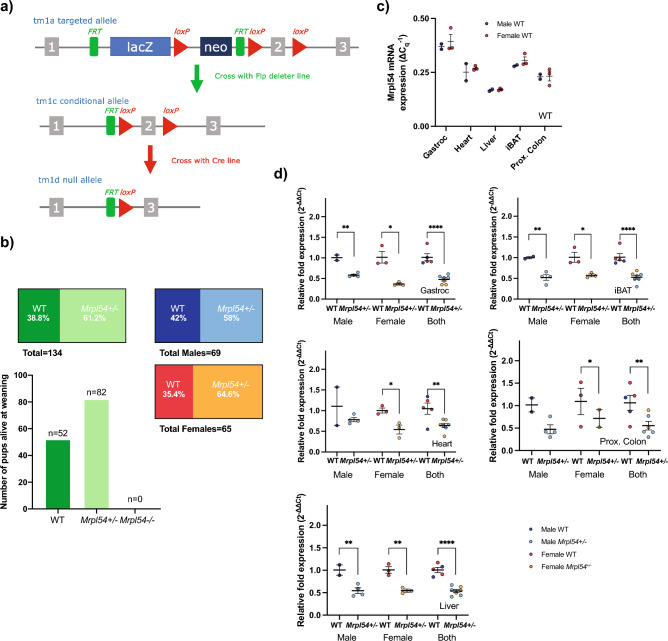
Generation and basic characterization of the *Mrpl54*^+*/−*^ model. (**a**) Generation of the *Mrpl54*^+*/−*^ mouse model through the deletion of exon 2 at the *Mrpl54* allele. Adapted from the Mouse Genome Database^61^. (**b**) The number of WT and *Mrpl54*^+*/−*^ pups born to 28 *Mrpl54*^+*/−*^ breeding pairs followed a Mendelian ratio for homozygous lethality*.* (**c**) Relative *Mrpl54* gene expression in tissues from 7-week-old WT males (blue, n = 2) and females (orange, n = 3) as determined by RT-qPCR. Values were normalized to 60S acidic ribosomal protein P0 (*36b4* or *RPLP0*) and beta-2-microglobulin (*B2m*) expression. (**d**) Reduced *Mrpl54* gene expression (~ 50%) in tissues derived from 7-week-old *Mrpl54*^+*/−*^ males (light blue, n = 2) and females (light orange, n = 3) compared to WT male (blue, n = 4) and female (orange, n = 3). Values were normalized to *36b4* and *B2m* expression. Graphs show mean ± SEM, *P ≤ 0.05, **P ≤ 0.01, and ****P ≤ 0.0001.

To investigate the effect of reduced *Mrpl54* expression on whole body metabolism and longevity, we generated mice heterozygous for *Mrpl54* (Fig. 1a) by deleting exon 2 in the *Mrpl54* gene. Homozygous *Mrpl54* exon 2 deletion proved lethal, as no *Mrpl54*^*−/−*^ mice were observed at weaning, yet WT and *Mrpl54*^+*/−*^ were born at the expected Mendelian ratio (Fig. 1b). This agrees with current understanding that there is little functional redundancy in *Mrp* genes^40^. Next, to assess if *Mrpl54*^+*/−*^ led to reduced expression of the targeted gene, we performed qPCR analysis in gastrocnemius, heart, liver, interscapular brown adipose tissue (iBAT), and proximal colon tissues from 7-week-old male and female mice. We found that the relative expression of *Mrpl54* was highest in gastrocnemius and lowest in the liver (Fig. 1c). Additionally, in all tissues tested, *Mrpl54* expression was reduced by ~ 50% in *Mrpl54*^+/−^ mice compared to WT (Fig. 1d).

### No major metabolic phenotype in adult Mrpl54^+/−^ mice

**Figure 2:**
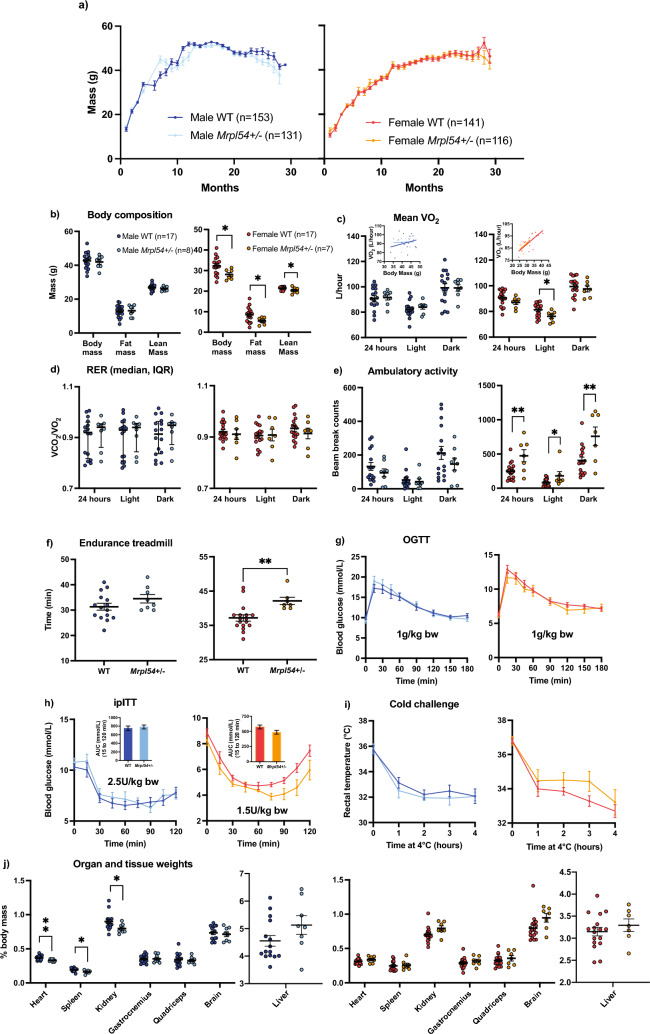
Metabolic phenotyping in 6-month-old male and female *Mrpl54*^+*/−*^ and wild type mice. (**a**) Body mass (g) comparison between male *Mrpl54*^+*/−*^ (n = 131) and WT (n = 153) and female *Mrpl54*^+*/−*^ (n = 116) and WT (n = 141) over lifespan. (**b**) Body composition (g) by EchoMRI in 6 M adult *Mrpl54*^+*/−*^ and WT male and female mice. (**c**) Mean VO_2_ (L/h) by CLAMS in 6 M adult mice. ANCOVA results (insets) indicate the difference in mean female VO_2_ are explained by the difference in female body mass. (d) Mean RER by CLAMS in 6 M adult *Mrpl54*^+*/−*^ and WT male and female mice. (**e**) Mean ambulatory motion (beam break counts) in 6 M adult *Mrpl54*^+*/−*^ and WT male and female mice. (**f**) Mean duration (min) on an endurance treadmill for 6 M adult male *Mrpl54*^+*/−*^ (n = 8) and WT (n = 15) male and *Mrpl54*^+*/−*^ and WT female mice. (**g**) Mean blood glucose levels (mmol/L) over time in response to an oral glucose challenge (OGTT) in 6 M *Mrpl54*^+*/−*^ and WT male and female mice. (**h**) Mean blood glucose levels (mmol/L) over time in response to intraperitoneal insulin challenge (ipITT) in 6 M adult *Mrpl54*^+*/−*^ and WT male and female mice with area under the curve (AUC) (insets). (**i**) Mean rectal temperature (°C) in response to a 4-h 4 °C cold challenge in 6 M male *Mrpl54*^+*/−*^ (n = 7) and WT (n = 16) mice and female *Mrpl54*^+*/−*^ and WT mice. (**j**) Relative organ weights (% body mass) in 6 M adult necropsied male *Mrpl54*^+*/−*^ (n = 7–8) vs. WT (n = 14–15) and female *Mrpl54*^+*/−*^ vs. WT mice. Graphs show mean ± SEM, *P ≤ 0.05, **P ≤ 0.01, and ****P ≤ 0.0001. Unless otherwise indicated, the number analysed are as follows: male WT (blue, n = 16); male *Mrpl54*^+*/−*^ (light blue, n = 8); female WT (orange, n = 17); and female *Mrpl54*^+*/−*^ (light orange, n = 7).

To examine the effect of reduced *Mrpl54* expression on metabolic health in adult mice, we subjected male and female *Mrpl54*^+/−^ and WT mice to a comprehensive metabolic phenotyping protocol (Supplemental Fig. S1). In both males and females in the combined longevity and metabolic study cohorts (the latter study animals included up until entering metabolic phenotyping, as described in “Methods”), there were no differences in body mass between *Mrpl54*^+/−^ and WT mice (Fig. 2a). Both genotypes had similar lean- or fat-mass body composition across genotypes in males; although, females in the metabolic health study at 6 months (6 M) had slightly lower lean and fat mass (Fig. 2b). Body composition as a percentage of body mass, however, remained the same between 6 M female *Mrpl54*^+*/−*^ and WT (Fig. S2). Metabolic indices, examined via indirect calorimetry, exhibited no differences in mean O_2_ volume (VO_2_) (Fig. 2c) or mean respiratory exchange ratio (RER) (Fig. 2d). Mean VO_2_ was lower in 6 M female *Mrpl54*^+/−^ animals during the less active light phase; however, an ANCOVA analysis^41^ attributed this difference to their reduced body mass (Fig. 2c inset). Male spontaneous ambulatory activity in testing cages was not different, yet there was an increase in activity in 6 M *Mrpl54*^+/−^ females when compared to WT (Fig. 2e), possibly in line again with the effect of reduced body weight and VO_2_. Like the increase in ambulatory activity of female *Mrpl54*^+/−^ mice, 6 M females exhibited improved running capacity in a forced exercise treadmill test (Fig. 2f). Whole body glucose handling was not different between genotypes during an oral glucose tolerance test (OGTT; Fig. 2g) or an intraperitoneal insulin tolerance test (ipITT; Fig. 2h) in 6 M male and female mice. Since skeletal muscle exhibited the highest relative levels of *Mrpl54* gene expression and iBAT tissue the second highest (Fig. 1c), and because of their importance for non-shivering and shivering thermogenesis, we evaluated potential differences in metabolic heat production by exposing mice to an acute cold challenge (4 °C). Mice from both genotypes maintained their core body temperature similarly for 4 h for 6 M males and females (Fig. 2i). At sacrifice, the masses of the heart, spleen and kidney were reduced as a percentage of body mass in *Mrpl54*^+/−^ males with no changes in skeletal muscle, brain, or liver (Fig. 2j). In females, there were no changes in organ masses expressed as a percentage of body mass. Overall, there were no robust morphological or metabolic changes in adult *Mrpl54*^+/−^ mice apart from the increased physical activity in female *Mrpl54*^+/−^ mice.

### Aged Mrpl54^+/−^ mice did not show improved metabolic phenotypes

**Figure 3:**
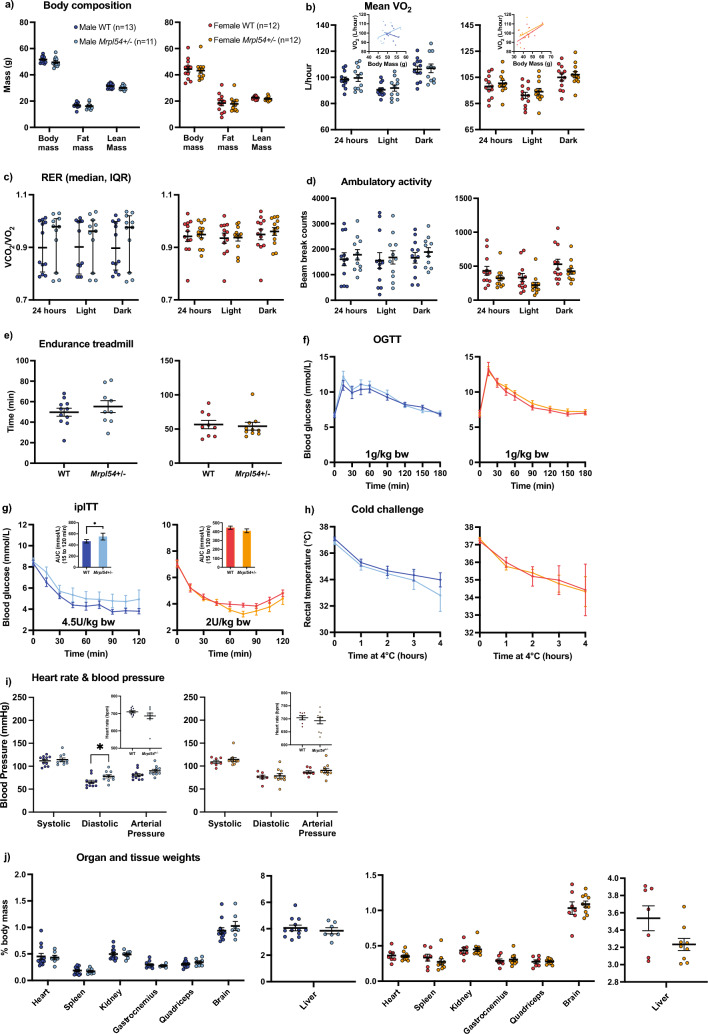
Metabolic phenotyping in 24 M *Mrpl54*^+*/−*^ and wild type mice. (**a**) Body composition (g) by EchoMRI in 24 M male *Mrpl54*^+*/−*^ (n = 12) and WT (n = 10) and female *Mrpl54*^+*/−*^ (n = 11) and WT (n = 13). (**b**) Mean VO_2_ (L/h) by CLAMS and body mass ANCOVA analysis (insets) in 24 M old male *Mrpl54*^+*/−*^ (n = 10) and WT (n = 9) and female *Mrpl54*^+*/−*^ and WT. (**c**) Mean RER by CLAMS in 24 M old male *Mrpl54*^+*/−*^ (n = 10) and WT (n = 9) and female *Mrpl54*^+*/−*^ and WT. (**d**) Mean ambulatory motion (beam break counts) in 24 M old male *Mrpl54*^+*/−*^ (n = 9) and WT (n = 9) and female *Mrpl54*^+*/−*^ (n = 10) and WT (n = 13). (**e**) Mean duration (min) on an endurance treadmill for 24 M old mice male *Mrpl54*^+*/−*^ (n = 6) and WT (n = 7) and female *Mrpl54*^+*/−*^ (n = 7) and WT (n = 9). (**f**) Mean blood glucose level (mmol/L) over time in response to an oral glucose challenge (OGTT) in 24 M old male *Mrpl54*^+*/−*^ (n = 10) and WT (n = 8) and female *Mrpl54*^+*/−*^ (n = 10) and WT (n = 12). (**g**) Mean blood glucose level (mmol/L) and AUC (insets) over time in response to intraperitoneal insulin challenge (ipITT) in 24 M old male *Mrpl54*^+*/−*^ (n = 8) and WT (n = 8) and female *Mrpl54*^+*/−*^ (n = 10) and WT (n = 11). (**h**) Mean rectal temperature (°C) in response to a 4-h 4 °C cold challenge in 24 M old male *Mrpl54*^+*/−*^ (n = 4) and WT (n = 3) and female *Mrpl54*^+*/−*^ (n = 4) and WT (n = 7). (**i**) Mean heart rate (beats/min) and blood pressure (mmHg) in 24 M old male *Mrpl54*^+*/−*^ (n = 6) and WT (n = 5) and female *Mrpl54*^+*/−*^ (n = 5) and WT (n = 8). (**j**) Relative organ weights (% body mass) in 24 M necropsied male *Mrpl54*^+*/−*^ (n = 4–5) and WT (n = 3–4) and female *Mrpl54*^+*/−*^ (n = 3) and WT (n = 6–7). Graphs show mean ± SEM; male WT (blue); male *Mrpl54*^+*/−*^ (light blue); female WT (orange); and female *Mrpl54*^+*/−*^ (light orange).

Given that disruption of mitochondrial translation and/or UPR^mt^ induction has been linked to improved healthspan in several animal models^15,30–35^, we examined whether reduced *Mrpl54* expression altered whole body metabolism during the aging trajectory. Female and male *Mrpl54*^+/−^ and WT mice were aged 24 months (24 M) before performing the same metabolic phenotyping tests used for adult 6 M mice. While there were no differences in body composition between *Mrpl54*^+*/−*^ and WT, female mice had increased fat mass and decreased lean mass with age, as a proportion of body mass, while the opposite was observed in males—fat mass decreased and lean mass increased with age, as a proportion of body mass (Supplemental Fig. S3, Fig. 3a). Like 6 M animals, we did not observe any differences in indirect calorimetry measurements between *Mrpl54*^+*/−*^ and WT at 24 M (Fig. 3b–d). At 24 M, there were no differences in running capacity (Fig. 3e), which contrasts our observations in 6 M *Mrpl54*^+*/−*^ female mice where running capacity was higher (Fig. 2f) but can be attributed to the difference in body weight at 6 M. In the 24 M mice, the response to OGTT was similar between *Mrpl54*^+*/−*^ and WT (Fig. 3f), as was the response to the ipITT (Fig. 3g). There were no differences between genotypes when exposed to a cold challenge at 24 M (Fig. 3h). There were no differences in heart rate, diastolic blood pressure, or systolic blood pressure between genotypes for either males or females at 24 M (Fig. 3i). Finally, at 24 M there were no differences in organ mass as a percentage of body mass (Fig. 3j). The 18 M cohort displayed the same results as the 24 M cohort for both males and females (Supplemental Fig. S4a–h, S4j); except 18 M male *Mrpl54*^+/−^ mice demonstrated a mean diastolic blood pressure that was higher than WT (Supplemental Fig. S4i). Altogether, aging *Mrpl54*^+/−^ mice did not reveal robust differences from WT in metabolic parameters.

### Lifespan and life expectancies were not altered in Mrpl54^+/−^ mice

**Figure 4:**
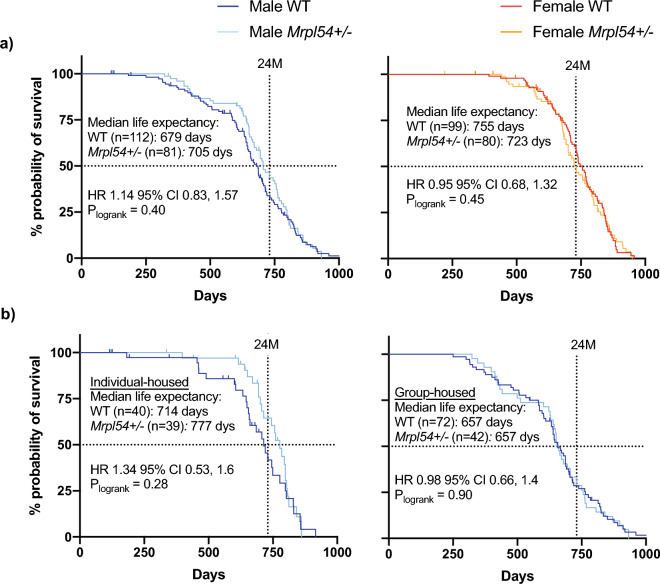
Life expectancy and lifespan in male and female *Mrpl54*^+*/−*^ mice. (**a**) Kaplan Meier survival curves showed no difference in overall lifespan (top 10% of surviving animals) between either male *Mrpl54*^+*/−*^ (n = 81) and WT (n = 112) or female *Mrpl54*^+*/−*^ (n = 80) and WT (n = 99) mice. No differences were observed in median life expectancy between *Mrpl54*^+*/−*^and WT for either males or females. (**b**) Kaplan Meier survival curves showed no difference in overall lifespan between either individual-housed male *Mrpl54*^+*/−*^ (n = 39) and WT (n = 40) or group-housed male *Mrpl54*^+*/−*^ (n = 42) and WT (n = 72). Individual-housed *Mrpl54*^+*/−*^ male mice had better median life expectancy compared to individual-housed WT, with no difference in median life expectancy between group-housed male *Mrpl54*^+*/−*^ and WT.

Since reduced MRP gene expression extended lifespan in *C. elegans* and correlated with long lifespan in BXD mouse strains, we tested whether reducing *Mrpl54* expression affected lifespan or life expectancy in mice. We followed *Mrpl54*^+*/−*^ male and female mice until they reached a humane endpoint or a natural death and assessed mean and 24-month life expectancy and maximum lifespan. We included 24-month life expectancy because strain-specific conditions and diseases affect C57BL/6 mice after the age of 24-months, which can confound results of aging studies^42^. We observed no significant differences in median life expectancy, 24-month life expectancy, or maximum lifespan between *Mrpl54*^+/−^ and WT, for either males or females (Fig. 4a).

During the lifespan study, some males were separated due to injuries or deaths from aggression and fighting, which resulted in a population of male mice that were housed individually (i.e., a single animal per cage from age 3–4 months onward). When individually and group-housed animals were analysed separately, it was evident that individually housed males had better median life expectancy than grouped-housed males for both genotypes (777 days vs. 714 days; Fig. 4b), which may be due to conspecific aggression increasing stress levels and shortening lifespan in group-housed males^43^. While not statistically significant, survival data for individually housed *Mrpl54*^+/−^ males suggested a better 24-month life expectancy compared to WT (24-month HR 1.85, 95% CI 0.86, 4.01, p = 0.12) (Supplemental Fig. S5a). Group-housed males (n = 2–9 males per cage) showed no differences in median or 24-month life expectancy between genotypes (Supplemental Fig. S5b).

### Mrpl54^+/−^ did not activate the UPR^mt^ in liver mitochondria or primary myoblasts

**Figure 5:**
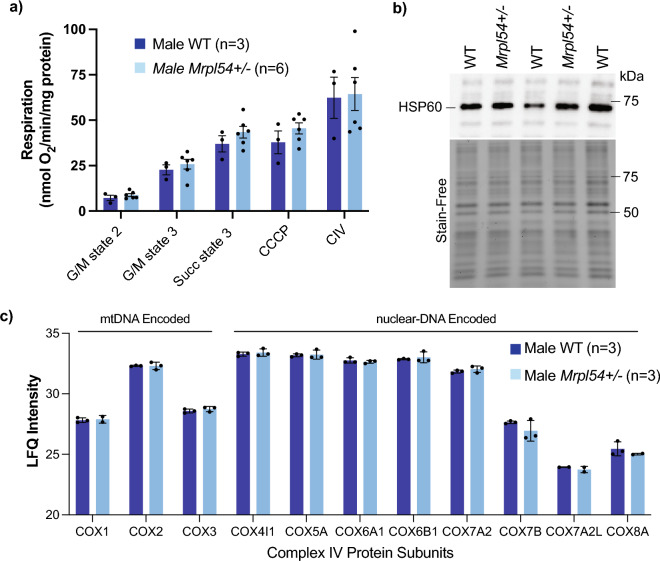
Biochemical profiling of *Mrpl54*^+ / −^ and wild type mouse liver. (**a**) Oxygraph respiration analysis of isolated liver mitochondria showed no difference in state 2, state 3, or maximal respiration between *Mrpl54*^+*/−*^ (n = 6) and WT (n = 3). (**b**) Semiquantitative western blot analysis of HSP60 expression in isolated liver mitochondria showed no difference between *Mrpl54*^+*/−*^ (n = 2) and WT (n = 3). (**c**) Mass spectrometry analysis of protein from isolated liver mitochondria showed no difference between levels (label-free quantification, LFQ) of nDNA- and mtDNA-encoded protein subunits of ETC complex IV between *Mrpl54*^+*/−*^ (n = 3) and WT (n = 3). Graphs show mean ± SEM; male WT (blue); male *Mrpl54*^+*/−*^ (light blue).

Since MRP proteins play a crucial role in the translation of mtDNA-encoded mRNAs into protein subunits for oxidative phosphorylation (OXPHOS), we determined whether mitochondrial oxygen consumption was affected in the liver, a major metabolic organ. Using a Clark-type oxygen electrode, purified mitochondria from the liver of 7-week-old WT and *Mrpl54*^+/−^ males demonstrated similar rates of oxygen consumption (Fig. 5a). We then examined if reductions in *Mrpl54* induced a mitonuclear protein imbalance and induced the UPR^mt^. Using purified mitochondria from the liver of 7-week-old WT and *Mrpl54*^+/−^ males, we found no significant difference in HSP60 (HSPD1) protein levels, a classical marker for UPR^mt^ activation^44,45^ (Fig. 5b, uncropped gel images in Fig. S6a). This result was confirmed by mass spectrometry-based proteomics on purified liver mitochondria from 7-week-old males showing no differences in the levels of nDNA- or mtDNA-encoded proteins between WT and *Mrpl54*^+/−^ animals (Fig. 5c). Combined, these results suggest that the mitochondrial translational capacity was not affected in the liver tissue of *Mrpl54*^+/−^ animals.

**Figure 6:**
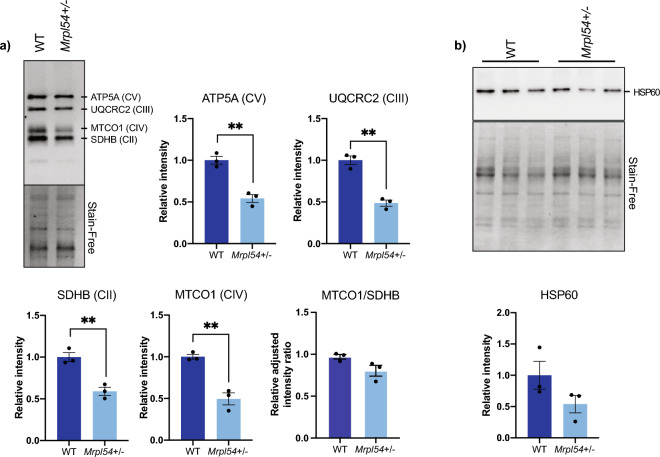
Biochemical profiling of *Mrpl54*^+*/−*^ and wild type mouse muscle. (**a**) Semiquantitative representative western blot of ETC nDNA-encoded subunits (ATP5A, QCRC2, and SDHB) and mtDNA-encoded MTCO1 subunit in cell lysate from primary myoblasts showed a decrease in ETC subunit expression in *Mrpl54*^+*/−*^ (n = 3) compared to WT (n = 3). (**b**) Semiquantitative western blot analysis of HSP60 expression in cell lysate from primary myoblasts showed no difference in HSP60 levels in *Mrpl54*^+*/−*^ (n = 3) compared to WT (n = 3). Graphs show mean ± SEM; male WT (blue); male *Mrpl54*^+*/−*^ (light blue); *P ≤ 0.05, **P ≤ 0.01, and ***P ≤ 0.001.

Since the relative *Mrpl54* expression is highest in skeletal muscle tissue (Fig. 1c), we examined the effect of reduced *Mrpl54* expression on the levels of OXPHOS protein subunits and the UPR^mt^ marker HSP60 in primary myoblasts. Primary myoblast cultures were established from 7-week-old male *Mrpl54*^+*/−*^ and WT hindlimb skeletal muscle. In cell lysates from these myoblasts, levels of mitochondrial OXPHOS subunits (including both nDNA- and mtDNA-encoded proteins) were lower in *Mrpl54*^+*/−*^ mice compared to WT (Fig. 6a, uncropped gel images in Fig. S6b,c). The level of succinate dehydrogenase complex iron sulphur subunit B (SDHB), a nDNA-encoded protein subunit of Complex II of the ETC, and the level of MTCO1, a mtDNA-encoded protein subunit of Complex IV of the ETC, were both lower in *Mrpl54*^+*/−*^ primary myoblasts (Fig. 6a, uncropped gel images in Fig. S6b,c). Notably, considering that both MTCO1 and SDHB were similarly decreased, the ratio of mtDNA- to nDNA-encoded proteins was not different in *Mrpl54*^+*/−*^ myoblasts. In line with this, HSP60 levels in *Mrpl54*^+*/−*^ myoblasts were not increased, suggesting that UPR^mt^ was not invoked in *Mrpl54*^+*/−*^ myoblasts (Fig. 6b, uncropped gel images in Fig. S6b). These results suggest that reduced *Mrpl54*^+*/−*^ expression in primary myoblasts lowered levels of various ETC complexes but this reduction was insufficient to activate the UPR^mt^.

## Discussion

Reduced expression of MRP proteins in *C. elegans* results in an increase in both healthspan and lifespan through the induction of the UPR^mt^ that results from a mitonuclear protein imbalance^15^. Similarly, long lifespan is correlated with low expression of *Mrps5* and other MRP genes in a genetic reference population of mice and elevated UPR^mt^ markers are observed in cell cultures of the long-lived Snell dwarf mouse strain^15,29^. The UPR^mt^ is also induced in mice treated with doxycycline^46^, an antibiotic that inhibits mitochondrial translation^47^, which potentially reduces energy expenditure^46^. To determine whether reduced expression of *Mrpl54* in heterozygote mice alters metabolism or lifespan, we compared metabolic health at 6-, 18-, and 24-months as well as assessed lifespan and life expectancy in *Mrpl54*^+/−^ and WT animals.

While *Mrpl54* mRNA expression was reduced in multiple metabolic organs from *Mrpl54*^+/−^ animals, we found no compelling metabolic changes in 6-month-old adult mice. However, in line with previous reports showing an increase in rearing activity during test cage monitoring in mice treated with doxycycline^46^, female *Mrpl54*^+/−^ mice exhibited an increase in ambulatory activity along with elevated treadmill endurance when compared to WT. Like doxycycline-treated mice^15,46^, we observed a lower VO_2_ in adult female *Mrpl54*^+*/−*^ mice, but this is likely explained by their reduced body weight^41^, and not consistent with observations in male mice, or female mice at later time points.

We compared the life expectancy and lifespan in large cohorts of *Mrpl54*^+/−^ and WT male and female mice. As a result of injury or death associated with male aggression, the male cohort was divided into individual-housed and group-housed cages. Male mice tended to experience stress associated with conspecific aggression under group housing conditions. Individually housed male *Mrpl54*^+*/−*^ mice had better median life expectancy than male WT mice, while group-housed males had no difference in median life expectancy between *Mrpl54*^+/−^ and WT. Male C57BL/6 mice can be successfully housed together in low numbers^43^; however, it may be that the high level of conspecific aggression experienced in male group housing overcame any survival advantage afforded by the *Mrpl54*^+/−^ genotype.

Finally, we observed neither a mitonuclear imbalance nor an induction of the UPR^mt^ in *Mrpl54*^+/−^ mouse liver, a major metabolic organ. Within primary myoblasts, however, there was evidence of decreased OXPHOS protein expression, but no evidence of UPR^mt^ induction. It remains to be seen whether there is an induction of the UPR^mt^ in other *Mrpl54*^+/−^ mouse tissues and organs, or in a temporal fashion—neither of which was captured in this study. Previous work demonstrated that reductions in MRP expression (through RNAi of MRP genes, or inhibition of mitochondrial translation through doxycycline treatment) can increase healthspan and lifespan in *C. elegans* through a mitohormetic response^15^. Our work demonstrates that a reduction in *Mrpl54* expression in the germline may have implications in energy expenditure and activity levels in adult female mice, along with a potential increase in median and 24-month life expectancy of individual-housed male mice that are removed from the conspecific stress associated with group housing conditions. However, our findings also demonstrate that a reduction of *Mrpl54* is not sufficient to drive the induction of UPR^mt^ in mouse liver tissue or primary myoblasts, nor is it able to alter metabolism of older mice or affect lifespan.

It may be that reducing *Mrpl54* expression through the mammalian germline allows the mouse to adjust to lower MRPL54 protein levels through adaptations during developmental processes from embryogenesis onward—for example, genetic compensation where mutant mRNA degradation leads to transcriptional adaptation^48^. In other words, the levels of MRP expression are adjusted early in development to optimize mitochondrial output and maintain mitochondrial protein homeostasis. Alternatively, our results may indicate that the level of mitochondrial translation stress instigated by the *Mrpl54*^+*/−*^ genotype is not high enough to generate a mitonuclear imbalance, mito-cytosolic translational imbalance, or induce mitochondrial stress responses like the UPR^mt^ or ATF4-dependent signalling^15,21,22^. This explanation implies that any mitohormetic effect that affects metabolic health or lifespan requires a mitochondrial translational stress level above a specific threshold, and that this threshold was not met by the *Mrpl54*^+*/−*^ model.

In conclusion, our results demonstrate that the reduction of *Mrpl54* expression through the mouse germline does not induce a mitonuclear imbalance or a subsequent UPR^mt^ response. Furthermore, reduction in *Mrpl54* expression does not result in an improved life expectancy or overall lifespan. We recommend that future work pursue the hypothesis of a minimum threshold of mitochondrial translational stress necessary to invoke a mitohormetic impact on metabolic health with age or lifespan. As well, since reduced *Mrpl54* expression in male mice may mitigate the lifespan effects of stressful housing conditions, we recommend that the *Mrpl54*^+*/−*^ model be tested under various stress conditions, such as genotoxic or metabolic stress through a high fat diet.

## Methods

### Approval for animal experiments

All procedures for animal care and experimentation were performed according to protocol HS-2847, which was approved by the University of Ottawa Animal Care Committee, and following guidelines provided by the Canadian Council on Animal Care (CCAC). In addition, these procedures followed the guidelines provided by ARRIVE (Animals in Research: Reporting In Vivo Experiments).

### Animal breeding

The Institute Clinique de la Souris (ICS) generated a *Mrpl54* constitutive knock-down mouse model on a C57BL/6N(Taconic) background (C57BL/6NTac-Mrpl54^tm1b(EUCOMM)Wtsi/IcsOrl^; colony name ICS-EPD1076_5_A09)^49,50^. *Mrpl54*^+/−^ breeding pairs were kept in ventilated racks and their progeny were housed in groups of 2–5 mice until their transfer to the metabolic health and longevity studies. Litter sizes were small (mean 4–5 pups) and production dropped off after the fourth litter. To generate the cohorts needed, CD1 host mothers were used for three rounds of in vitro fertilization, in addition to traditional breeding. At age 21-days, animals were weaned, ear-tagged, and tissue sampled from the ear for genotyping.

### Genotyping

DNA for genotyping was extracted from the ear tissue sample according to the Phire Animal Direct PCR protocol (F-170, Thermo Scientific). Genotyping proceeded according to the ICS protocol (Forward primer Ef 4877 5′-GACCCACATAAGCAGGGAAGGAGATG-3′, reverse primer L3r 4879 5′-CAATCTCCTGAGAATGTAGCCCACCAT-3′, Invitrogen). The *Mrpl54* knock-out allele generated a 402 base-pair (bp) fragment, and the WT allele generated a 1095 bp fragment.

### Animal husbandry

WT and *Mrpl54*^+/−^ animals, separated by sex, were housed under a 12:12-h light–dark cycle in a dedicated room set to 23 °C (temperature and humidity were checked every 5 min using an automatic delta system; verified daily by staff). All mice were fed a standard chow diet (Envigo; Teklad Global 2018; 18.6% crude protein, 6.2% fat, 44.2% available carbohydrates). Female and male mice were initially housed 5–10 per cage in a ventilated rat cage (at least 90.4 cm^2^ per mouse) to reduce stress^51^. Male cages experienced aggression and fighting and the male mice in cages that experienced injuries or deaths due to male-on-male aggression were reassigned to individual-housing. Animals in the metabolic health study were handled or weighed at least once per week. Animals in the longevity study were weighed once per month until age-related weight loss was observed, after which mice were weighed weekly or daily as needed.

### RNA isolation and real-time qPCR

For total RNA isolation, a small piece of snap frozen mouse tissue was placed in 1 ml TRIzol (Sigma-Aldrich or Invitrogen). Samples were homogenized with a 5 mm steel bead using a TissueLyser II (Qiagen) for 3 min at a frequency of 30 Hz. RNA was isolated according to manufacturer’s protocol. From 1 μg of extracted RNA, genomic DNA was eliminated with a gDNA Wipeout Buffer and subsequently reverse transcribed into cDNA using the QuantiTect Reverse Transcription Kit (Qiagen). The diluted cDNA was prepared with SYBR Green (Roche), and the quantitative gene expression was measured using the LightCycler 480 Instrument (Roche). The primer pairs used were as follows: *Mrpl54* forward 5′-AAAAAGCCAGTTGGCAAGGG-3′, reverse 5′-ATGTGTGGTGAGCTGAGTGG-3′; *36B4* (acidic ribosomal phosphoprotein gene) forward 5′-ACGGGTACAAACGAGTCCTG-3′, reverse 5′-GCCTTGACCTTTTCAGCAAG-3′; and *B2m* (beta2 microglobulin) forward 5′-GGCTCACACTGAATTCACCC-3, 5′-GTCTCGATCCCAGTAGACGG-3′. To normalize tissue sample expression (threshold cycle [Ct] values) to the expression of housekeeping genes, the delta-delta Ct method was used. The geometric means of *36B4* and *B2m* genes were used to normalize *Mrpl54* tissue sample expression (delta Ct values) to the WT samples.

### Metabolic health analyses

The metabolic health of male and female WT and *Mrpl54*^+/−^ mice (n = 7–17 in each group) were tested in three separate cohorts at 6-, 18-, and 24-months of age. The battery of tests started with EchoMRI body composition (EchoMRI-700 Analyzer), followed by indirect calorimetry using the comprehensive laboratory animal monitoring system (CLAMS, Columbus Instruments), oral glucose tolerance, intraperitoneal insulin tolerance, endurance treadmill, cold tolerance, heart rate and blood pressure measurement (at 18- and 24-months), and then necropsy (Supplemental Fig. S1). Mice were allowed to recover 1–2 weeks between tests.

### EchoMRI and indirect calorimetry

Each mouse was loaded into an A100 antenna insert and placed into the EchoMRI (EchoMRI-700 Analyzer) to determine body composition (whole-body, lean, and fat mass). Immediately afterward, mice were placed in individual CLAMS (Columbus Instruments Oxymax System) plexiglass cages for 48–72 h (24-h acclimation, followed by 24–48 h of data collection). The ambient temperature was set to thermoneutrality (28 °C) to gain an understanding of basal metabolism that mimicked human energy expenditure and to reduce thermogenic stress on mice housed individually without bedding or enrichment^52^. The light–dark cycle was 12:12. Mice had ad libitum access to water and powdered chow (Teklad 2018 Diet). At regular intervals (every 18–26 min), the following measurements were recorded: O_2_ volume (VO_2_), CO_2_ volume (VCO_2_), respiratory exchange ratio (RER), ambulatory and rearing motion, and the amount of food consumed in grams.

### Oral glucose tolerance test (OGTT)

Mice were fasted overnight for 16-h prior to the OGTT. Whole-blood glucose levels were measured using the Accu-check Performa glucometer and accompanying strips (Roche Diagnostics, Canada). For the 6 M cohort, mice were placed in a restraint tube during the blood sampling from the left lateral tail vein. For the 18 M and 24 M cohorts, mice remained unrestrained on the home-cage hopper during blood sampling. At time 0, freshly made 20% α-d-glucose (Sigma-Aldrich PN158968) was orally administered using a flexible straight metal gavage at a dose of 1.0 g per kg of body mass. Blood glucose was measured at baseline (prior to oral glucose dose), and at 15-, 30-, 45-, 60-, 90-, 120-, 150-, and 180-min post-gavage.

### Intraperitoneal insulin tolerance test (ipITT)

Two weeks prior to the ipITT, intraperitoneal insulin titrations were conducted on a subset of mice to determine the correct dose to elicit an insulin response. Mice were fasted in the morning for 5–6 h prior to the ipITT. Whole-blood glucose levels were measured using the Accu-check Performa glucometer and accompanying strips (Roche Diagnostics, Canada). During blood sampling from the right lateral tail vein, mice remained unrestrained on their home-cage hopper. At time 0, mice received an intraperitoneal injection of insulin (NovoRapid Insulin aspart, 100U/mL, DIN 02245397). For the 6 M cohort, females received an insulin dose of 1.5 U/kg body mass and males received 2.5 U/kg. For the 18 M cohort, females received an insulin dose of 2 U/kg and males received 4.5 U/kg. For the 24 M cohort, females received an insulin dose of 1 U/kg and males received 2 U/kg. Blood glucose was measured at baseline (prior to insulin injection), and at 15-, 30-, 45-, 60-, 75-, 90-, 105-, and 120-min post-injection. In the event the blood glucose level dropped below 2.0 mmol/L, emergency α-d-glucose (Sigma-Aldrich PN 158968) was orally administered at a dose of 1 g per kg of body mass.

### Treadmill

Mice were acclimated to the treadmill (Exer-3/6, Columbus Instruments) over three consecutive days where they were placed on an unmoving belt for 5 min, then ran at a constant speed of 15 cm/s at an elevation of + 5° with an electro-stimulus (0.1 mA, 1 Hz) applied to the resting grid. Those mice that achieved fewer than 5 electro-stimulations over 5 min by the third acclimation day were permitted to continue with the endurance test. The endurance test was conducted the day following the last day of acclimation using the following profile—accelerate to 15 cm/s in 30 s and then run at 15 cm/s for 12 min, accelerating + 3.0 cm/s over 30 s every 12 min^53^. Mice ran until the exhaustion criterion was met (5 electro-stimulus contacts [0.1 mA, 1 Hz] within 5 s) at which point the electro-stimulus was stopped, and the running time and maximum speed attained were recorded.

### Cold tolerance

The baseline rectal temperature of each mouse was measured using a lubricated rectal thermometer at an insertion depth ~ 2 cm. Mice were placed in individual cages with access to fresh water and a plastic house, but without access to bedding or food, and then placed in a + 4 °C refrigerated room with all cages at the same height. Rectal temperature was measured every hour for up to 4–6 h. If rectal temperature reached 25 °C or below^54^, the mouse was removed from the cold room and placed in a 37 °C incubator with access to food and water.

### Heart rate and blood pressure

Mice in the 18 M and 24 M cohorts were acclimated to the blood pressure and heart rate monitoring equipment (BP-2000-M-6 Series II Blood Pressure Analysis System, Visitech Systems) over a period of 3–5 days^55^. The procedure for acclimation and testing was the same—mice were individually placed in an opaque mouse holder on a warmed 36 °C plate with the tail pulled through an inflatable tail cuff. Mice rested in position for 5 min, followed by 5 preliminary readings and then 10 actual readings. The program parameters were as follows: 2.5-s pause between readings, 10-s analysis pulse, system maximum set to 170 mmHg. Test day immediately followed the last day of successful acclimation, where blood pressure was recorded for at least 6 out of 10 readings. The outcomes recorded were diastolic blood pressure, systolic blood pressure, and heart rate.

### EchoMRI and necropsy

The day before the necropsy, body composition was determined using the EchoMRI-700 as described above. The mice were fasted overnight for 10–12 h and then refed 1–2 h before the necropsy. Mice were euthanized through injection with ketamine/xylazine cocktail (0.1 mg/kg body weight) followed by exsanguination through cardiac puncture. The following tissues and organs were collected, weighed, fast-frozen in liquid N_2_ and then stored at − 80 °C for further analysis: pancreas, spleen, diaphragm, heart, liver, kidneys, quadriceps, gastrocnemius, soleus, tibialis anterior, and brain^56^. For the 24-month cohort necropsy, the right kidney, quadriceps, and gastrocnemius/soleus were suspended in Optimal Cutting Temperature fixative (Fisher Health Care, Cat# 4585) and placed on dry ice (kidney) or in isopentane (Sigma, CAT# 1003301470) supercooled in liquid N_2_ (muscles), then stored at − 80 °C for further analysis.

### Longevity study and growth

Male and female WT and *Mrpl54*^+/−^ mice (n = 80–112 per group) were initially group-housed 5–10 per cage (at least 100 cm^2^ per mouse). Male cages experienced aggression and fighting from age 3-months onward. In case of a fight-related injury or death, the males were reassigned to individual cages to improve animal welfare. Mice were weighed monthly, and median life expectancy (50% survival time point), 24-month life expectancy (all mice alive at 24 months were censored), and maximum lifespan (lifespan of top 10% of surviving animals) were calculated. Where a human-endpoint (HEP) was identified and time-permitting, mice were necropsied to identify tumour burden and to collect tissue and plasma for future analysis. HEPs included (a) > 15% weight loss over 2 weeks, (b) skin lesions not responding to treatment, (c) head tilt/rolling or loss of balance, (d) ulcerated or bulging eye, (e) ulcerated mass, (f) respiratory distress, (g) inability to express bladder, or (h) a distended abdomen or growing internal mass^51^. Mice that were euthanized because of ulcerative dermatitis that did not respond to treatment (a C57BL/6 strain-specific condition^57^), or because of an unacceptable pain level from an ulcerated or bulging eye, were censored from the longevity analysis.

To assess growth over time, the body mass of WT and *Mrpl54*^+/−^ male and female animals were weighed monthly. Animals destined for the 6-, 18-, and 24-month metabolic health analyses were included in the growth analysis, up until the month they entered the metabolic health study.

### Respiration of isolated liver mitochondria

7-Week-old male WT and *Mrpl54*^+/−^ mice were fasted overnight for 12 h, killed by cervical dislocation, and their livers weighed then rapidly dissected directly into ice-cold isolation buffer (300 mM D-sucrose, 10 mM Tris–HCl, 1 mM EGTA-Tris Base; pH 7.2)^58^. The suspension was homogenized in a 15 mL Potter–Elvehjem homogenizer, and the supernatant centrifuged twice at 1000* g* for 10 min at 4 °C (Sorvall RC-6 Plus Centrifuge, Thermo Scientific). The supernatant was centrifuged 8000* g* for 10 min at 4 °C and then aspirated without disturbing the pellet. The pellet was gently resuspended in ice-cold suspension buffer (300 mM D-sucrose, 10 mM Tris–HCl, 0.05 mM EGTA-Tris Base; pH 7.2) and centrifuged at 8000* g* for 10 min at 4 °C. The final pellet was gently re-suspended in 300 µL suspension buffer and kept on ice.

The respiratory function of isolated liver mitochondria was measured using a Clark-type electrode (Oxygraph System, Hansatech Instruments Ltd.). The chambers were calibrated at 23 °C with continuous stirring with a PTFE-coated magnetic follower bar. The mitochondrial suspension was quantified (Bio-Rad DC Protein Assay, BMG Labtech POLARstar Omega plate reader) and then suspended in miR05 respiration buffer (110 mM D-sucrose, 60 mM lactobionic acid, 20 mM taurine, 20 mM HEPES, 10 mM KH2PO4, 3 mM MgCl2, 0.5 mM EGTA, 1 g/L fatty-acid free BSA; pH 7.1 at 23 °C)^59^ at 0.5 mg protein/mL. The function of isolated liver mitochondria was examined at less than 37 °C to prolong mitochondrial activity and maximize the duration of the experiment, such that O_2_ did not become rate-limiting^60^. Following a baseline recording, O_2_ consumption rate was measured following sequential additions of: (1) glutamate + malate (G/M, 5:2.5 mM) to give Complex I (CI)-driven State 2 respiration, (2) ADP (2 mM) to give CI-driven State 3 respiration, (3) Amytal (2 mM) to inhibit CI, (4) succinate (10 mM) to give Complex II (CII)-driven State 3 respiration, (5) carbonyl cyanide m-chlorophenylhydrazone (CCCP) titrations to decouple OXPHOS until maximum respiration was reached (0.01–0.05 µM); (6) antimycin-A (8 µM) to inhibit Complex III (CIII); (7) *N*,*N*,*N*′,*N*′-Tetramethyl-p-phenylenediamine dihydrochloride (TMPD) + ascorbate (5:0.3 mM) to test Complex IV (CIV) respiration; and finally 8) KCN (0.6 mM) to inhibit CIV.

### Mass spectrometry (MS)-based proteomics

Crude mitochondria were isolated from the livers of WT (n = 3) and *Mrpl54*^+/−^ (n = 3) male mice, as described above, and then purified using 30% Percoll gradient ultracentrifugation at 95,000*g* for 30 min at + 4 °C (Beckman sv41 rotor). Purified mitochondria were treated 1:200 with a protease inhibitor cocktail (Thermo Fisher Scientific HALT PI) then the mitochondrial proteins were left to precipitate overnight in precipitation buffer (50% acetone, 49.9% ethanol, 0.1% acetic acid). Proteins were suspended in 50 mM ammonium bicarbonate in 8 M urea and then quantified (Bio-Rad DC Protein Assay, BMG Labtech POLARstar Omega plate reader). To reduce disulphide bonds, 140 µg of mitochondrial protein was incubated with 1:100 of 1 M dithiothreitol (Sigma-Aldrich, SKU 43815), followed by 1:50 dark incubation in 1 M iodoacetamide (Sigma-Aldrich, SKU I1149). The urea concentration was reduced to < 2 M and the protein sample was digested overnight at 37 °C with 1:100 Trypsin Gold (Promega V5280). The following day, the digested proteins were passed through a Sep-Pak Vac 1 cc tC18 cartridge (Waters, WAT054960) to rid the sample of salts and contaminants. The sample was dried in a Savant DNA120 SpeedVac Concentrator (Thermo Electron Corporation) at room temperature and analysed using a nano-LC–MS/MS with an Orbitrap Elite mass spectrometer (Thermo Fisher Scientific) coupled to an ultraperformance LC system (Ultimate 3000 RSLC; Thermo Fisher Scientific). Data analysis was performed with Proteome Discoverer (version 1.3), and searches were performed with Mascot and Sequest against a mouse database (UniProt). Data were further processed, inspected, and visualized with Scaffold 4 (Proteome Software).

### Generation of primary myoblasts

Hind limb skeletal muscles were excised from euthanized 7-week-old male WT (n = 3) and *Mrpl54*^+/−^ (n = 3) mice. Under aseptic conditions, the muscles were rinsed in phosphate buffered solution (PBS, Wisent Inc., Cat# 311-010-CL), the fat excised, and the remaining muscle tissue placed in collagenase B-dispase solution, 1.5 mL per 0.5 g tissue (4 mg/mL dispase II [Sigma D4693], 10 mg/mL collagenase B [Roche 11088831001] in Hams-F10 [Multicell, Wisent Inc. Cat# 318 050-CL]). The tissue was mulched with a razor blade, incubated at 37 °C for 30 min, and then homogenized through trituration until smooth. The homogenate was passed through a 100 micron cell strainer with PBS washes. The muscle cells were centrifuged at 300* g* for 5 min and the cell pellet resuspended in Ham’s Complete growth medium (4% bovine calf serum [cytiva, Cat# SH30077.03], 1% penicillin and streptomycin [gibco by Life Technologies, Cat# 15140122], 2.5 ng/µL human basic fibroblast growth factor [bFGF, Stemcell, Cat# 78003]). To rid the culture of fibroblasts, the cells were allowed to rest on a non-collagen-coated plate for 2 h at 37 °C before plating onto a 10 cm collagen-coated plate. To enrich myoblasts, 80% confluent plates were pre-plated on non-collagen-coated plates for 1 h before plating on a collagen-coated plate.

### Western blotting

Protein was extracted by suspending isolated WT and *Mrpl54*^+/−^ liver mitochondria or primary myoblast cells (10 cm plates at 80% confluence) in radioimmunoprecipitation (RIPA) lysis buffer (50 mM Tris HCl, 150 mM NaCl, 1% v/v Triton X-100, 0.5% w/v sodium deoxycholate, 0.1% w/v SDS; pH 8.0) augmented with protease and phosphatase inhibitors (1:100 HALT PI cocktail, Thermofisher Sci Cat# 78440; or Roche cOmplete ULTRA [Cat# 05892970001] plus phosStop [Cat# 4906845001] tablets), then flash frozen in liquid N_2_. The solution was centrifuged 16,000*g* for 10 min at 4 °C and the supernatant containing the mitochondrial or cell lysate proteins quantified (Bio-Rad DC Protein Assay, BMG Labtech POLARstar Omega plate reader).

Protein samples were diluted to 1 µg/µL in Laemmli Buffer (Bio-Rad Cat# 1610737) augmented with 10% β-mercaptoethanol (Fisher Bioreagents, BP176-100) and run on a 10% SDS-PAGE using the TGX Stain-free FastCast Acrylamide Kit (Bio-Rad Cat# 1610183) and the Mini-PROTEAN system (Bio-Rad Cat# 1658006). SDS-PAGE was run at a constant 90 V across the gel for 30 min and then 120 V for 50–60 min in a 4 °C cold room. The StainFree gels were activated once for 45 s using a ChemiDoc Touch Imaging System (Bio-Rad) and then the proteins transferred to TransBlot Turbo Mini size 0.2 µm nitrocellulose membrane (Bio-Rad) using the Trans-Blot TurboTransfer System (Bio-Rad Cat# 1704150EDU).

The nitrocellulose membrane was imaged for total protein and then blocked for 1 h in blocking buffer (5% w/v bovine serum albumin [Sigma, SKU A7906] in TBS-T buffer [50 mM Tris–HCl, pH 7.6; 150 mM NaCl; 0.1% Tween]), rocking gently at room temperature. Membranes were then incubated with primary antibody diluted in TBS-T buffer (1:1000 Total OXPHOS Rodent WB Antibody Cocktail [abcam Cat# ab110413] or 1:1000 HSP60 XP Rabbit mAB [Cell Signaling Technology CAT#12165]) overnight at 4 °C with gentle rocking. After three washes with TBS-T, the membrane was incubated for 1 h with the matching IgG, horseradish peroxidase (HRP)-conjugated secondary antibody (1:10,000 Cell Signaling Technology Anti-rodent Cat# 7076, or Anti-rabbit Cat# 7074P2), rocking at room temperature. After three washes in TBS-T, the membrane was visualized using enhanced chemiluminescent detection (BioRad Clarity Cat# 1705061) on the ChemiDoc Touch Imaging System (Bio-Rad).

### Statistical analysis

All data are presented as mean ± SEM, unless otherwise stated. Differences between two groups were assessed using two-tailed t-tests. Analysis of covariance (ANCOVA) was used to eliminate unwanted variance on the dependent variable. To compare the interaction between time and treatment, a two-way analysis of variance (ANOVA) tests was performed. Survival curves were analysed using the Kaplan–Meier method. Hazard ratios were calculated using the Log rank test. GraphPad Prism 9.4.1 (GraphPad Software, Inc.) was used for all statistical analyses, and *p* < 0.05 was considered significant.

## Supplementary Information

Supplementary Figures.

## Data Availability

The datasets generated during and/or analysed during the current study are available from the corresponding author on reasonable request.
